# Prognostic impact of PTK6 expression in triple negative breast cancer

**DOI:** 10.1186/s12905-023-02736-y

**Published:** 2023-11-07

**Authors:** Yuexia Chen, Wei Qu, Jianhong Tu, Liu Yang, Xingxing Gui

**Affiliations:** Department of Pathology, Nanchang People’s Hospital(formerly The Third Hospital of Nanchang), No.1268 Jiuzhou Street, Chaoyang New City, Nanchang City, 333000 Jiangxi China

**Keywords:** Triple negative breast cancer, PTK6, DFS, OS, HER2 low expression

## Abstract

**Background:**

The aim of this study was to investigate the expression of PTK6 in different groups of triple negative breast cancer and its impact on prognosis.

**Methods:**

Retrospective study of a total of 209 surgical specimens of breast cancer were identified by IHC or FISH methods as triple negative,and divided into a lymph node metastasis positive (LNM +)group (*n* = 102) and a lymph node metastasis negative(LNM-) group (*n* = 107) according to the lymph node status of the surgical specimen. PTK6 expression was detected by IHC technique in all surgical specimens. PTK6 expression and clinicopathological features was explored by Chi-square test. The prognosis of different groups of patients was analyzed by Kaplan–Meier survival analysis and COX analysis.

**Results:**

The incidence of PTK6 expression in the LNM + group (78.4%) was significantly higher than in the LNM- group (28%). Clinicopathological analysis showed that PTK6 expression in the LNM + group was negatively correlated with the 5-year survival of patients. Kaplan–Meier analysis showed that only PTK6 expression in the LNM + group was negatively correlated with OS and DFS. COX analysis also showed that PTK6 expression and N stage were independent prognostic factors for DFS in the LNM + group. No correlation was observed between HER2 and PTK6 expression in any of the groups.

**Conclusions:**

This study suggests that PTK6 promotes tumor development and was associated with poor prognosis in the LNM + group of triple negative breast cancer. Inhibition of PTK6 may be a new approach for the treatment of triple negative breast cancer patients, especially those with metastasis.

## Introduction

Triple negative breast cancer (TNBC) is a subtype of breast cancer in which patients are at high risk of recurrence or metastasis despite receiving standard treatment.The poor prognosis of these patients means it is imperative to develop new therapeutic options. The current molecular understanding of TNBC metastasis is incomplete, and there is no standardized treatment in clinical practice [1]. It has been demonstrated that BRK/PTK6 is a central molecule in the regulation of numerous signals related to cell growth, proliferation, angiogenesis, survival, invasion, metastasis, apoptosis, and autophagy. This protein is highly expressed during the development and progression of breast cancer, and hence may serve as a target for breast cancer therapy [2, 3].

Protein tyrosine kinase 6,PTK6 (BRK, breast tumor kinase), located at 20q13.33, is a non-receptor kinase that consists of an amino-terminal domain, Src homologous SH3 domain, SH2 domain, and a carboxy-terminal tyrosine kinase catalytic domain [4–7]. The SH2 domain promotes proliferation and metastasis of tumor cells, however the impact of the kinase activity on tumor prognosis is controversial [8–10]. PTK6 is highly expressed in many tumor types, including lung, breast, ovarian and prostate cancers, as well as in about 70% of TNBC [11–14]. It has been reported that PTK6 promotes cell proliferation and migration by phosphorylating Eps8 [15]. In a TNBC cell model, PTK6 was found to regulate the expression of E-cadherin through the degradation of SNAIL protein, thereby promoting epithelial stromal transformation and regulating the migration of cells [14]. Brk/ptk6 is also an important downstream effect or of the Met signaling pathway in cell migration [16]. Therefore, targeting of PTK6 may serve as a therapeutic strategy for metastatic breast cancer and other cancer types.At the same time, many compounds have also been proved to inhibit the tumor process by inhibiting the expression of PTK6, such as (E)-5-(benzylideneamino)-1 h-Benzo [d]imidazol-2(3H), semi-synthetically optimized sipholenol A ester, and 4-aniline-substituted α-carboline compounds [17–19].

Most of the studies to date on PTK6 and breast cancer have been based on animal models or celllines, with little validation carried out in tumor tissues from breast cancer patients [14–16, 20]. Therefore, we collected surgical excision specimens from 209 patients with TNBC, of which 102 cases had lymph node metastasis (LNM +) and 107 cases did not (LNM-). We then conducted a five-year postoperative follow-up study to investigate the relationship between PTK6 expression and the metastasis, clinicopathological features and prognosis of TNBC.

## Materials and methods

### Case data

Specimens of 209 patients with invasive breast cancer who underwent mastectomy in the Department of Pathology, Nanchang People's Hospital(formerly The Third Hospital of Nanchang) from January 2013 to December 2017 were collected for retrospective analysis. Inclusion criteria: 1. All patients were pathologically confirmed to be invasive breast cancer; 2. Triple-negative breast cancer confirmed by immunohistochemistry or FISH; 3. The patient had complete clinicopathological data, postoperative pathology and follow-up data; Exclusion criteria: 1. Bilateral breast cancer patients; 2. Patients with other malignant tumors; 3; Preoperative radiotherapy, neoadjuvant chemotherapy and endocrine therapy, etc. 4. Patients who died due to accidents (such as traffic accidents or other sudden diseases) in the follow-up data.A total of 209 patients with TNBC were identified according to their negative ER, PR and HER2 status, as determined by IHC or FISH methods. Of these, 102 patients were LNM + and 107 patients were LNM-, according to the lymph node metastasis score of the surgical specimen. Patients with lymph node metastasis at the time of surgical removal of breast cancer specimens were identified as the LNM + group, and vice versa, as the LNM- group.According to the time of surgery for each patient, 5 years was set as the cut-off point, and 5-year overall survival (OS) and disease-free survival (DFS) were determined by telephone follow-up or imaging data.All patients provided written informed permission.

### Immunohistochemical(IHC) Test

Experimental reagent: The EnVision two-step method was used for IHC.Antibody kits for estrogen receptor (ER, clone number SP1) and progesterone receptor (PR, clone number SP2) were purchased from Fujian Maixin Company. Primary antibody for HER2 was purchased from Roche(VENTANA anti-HER2/nue(4B5)Rabbit Monoclonal Primary). PTK6 antibody (abs117941) was purchased from Uning Wei Co., LTD.Secondary antibody (MaxVision-HRP rat/rabbit) and DAB(3, 3-diaminobenzidine) color developing solution were purchased from Fuzhou Mai Xin Co., Ltd.

IHC staining procedure: surgically resected specimens were fixed with 10% neutral formalin, embedded in dehydrated paraffin, sliced continuously at 4 μm thickness, dewaxed, hydrated, washed with distilled water 3 times for 3 min each, and washed with PBS 3 times for 5 min each. The sections then underwent antigen repair with EDTA (pH9.0) and high-pressure steam for 3 min, and were cooled at room temperature. Wash 3 times with PBS for 5 min each; add3%H_2_0_2_ for 10 min; rinse 3 times with PBS for 5 min each; pour off the sealing solution and rinse 3 times with PBS for 5 min each. The staining procedures for ER, PRand HER2 were carried out in strict accordance with instructions from the antibody supplier. The incubation with ER and PRprimary antibody was for 30 min at room temperature, while the PTK6 antibody was diluted at 1:400 and placed in the refrigerator at 4 °C overnight. Drop plus primary antibody was placed at 4℃ overnight and washed 3 times with PBS for 5 min each. The secondary antibody (Maixin Company) was then added and allowed to stand at room temperature for 45 min; wash 3 times with PBS for 5 min each; DAB (3, 3-diaminobenzidine) color development kit (Maixin Company); hematoxyl in stained and sealed by dehydration.

Result Interpretation: (1) Interpretation of PTK6. Multi-head optical microscopes were used to grade staining results based on immune response scores (IRS) [21]. Staining intensity scores were 0(no staining), 1(weak staining, yellowish brown), 2(moderate staining, yellowish brown) or 3(strong staining, brown), while the percentage of positive cells was evaluated as 0(0%), 1(1–10%), 2(11–50%), 3(51–70%) or 4(71–100%). The score for PTK6 expression was obtained by multiplying the staining intensity with the number of stained positive cells. The median score (4.0) was chosen as the critical value. "PTK6-negative" was defined as a score of 0–4, and "PTK6-positive" as a score of 5–12. The stained tissue sections were evaluated and scored by two senior pathologists, neither of whom knew the clinical parameters.

(2) The interpretation of HER2 staining was according to the Breast Cancer HER-2 Detection Guidelines (2019 edition) [22]: 0: none of the cancer cells had any staining of the cell membrane; 1 + : any proportion of cancer cells showing weak or incomplete cell membrane staining; 2 + : > 10% of cancer cells showed mild to moderate, complete but uneven cell membrane staining, or < 30% of cancer cells showed strong and complete cell membrane staining; 3 + : ≥ 30% of cancer cells shows trong and intact cell membrane coloration.

(3) IHC positive for ER and PR was defined as ≥ 1% tumor cell nuclei showing different degrees of staining. Negative ER and PR were defined as < 1% tumor cell nuclei with varying degrees of staining or no staining [23].

### Fluorescence in situ hybridization (FISH)

The HER2 gene probe kit and protease were purchased from Wuhan Kanglu Co., Ltd. Methods: The 4 μm slices were heated overnight at 60℃, dewaxed, dehydrated in a gradient, boiled in distilled water for 30 min, digested with protease at 37℃, then denatured at 83℃ for 5 min, hybridized overnight at 42℃, washed and dried, then re-stained with DAPI and observed by fluorescence microscopy.

Result interpretation:HER2 gene signal was shown in orange, and the centromeric signal on chromosome 17 was shown in green. A total of 100 cancer cells were counted, the total value of orange signal and green signal was counted, and the ratio calculated. A ratio < 2.0 was classified as negative and considered as no gene amplification, while ratios > 2.0 or clusters of orange signals were classified as positive and considered as gene amplification.If the ratio was 1.8 to 2.0, the cell count was increased to 200, or the test was repeated.

### Statistical methods

Statistical analysis was performed by SPSS 22.0 software and Graphpad 9.0 software. The difference of measurement data was analyzed by T test in Graphpad 9.0 software.Chi-square test and the Pearson Chi-Square,Continuity Correction and Fisher's Exact Testmethods were used to analyze the correlation between the expression of PTK6 in different groups and the clinicopathological characteristics of patients. The Kaplan–Meier method was used for survival analysis and the log-rank test to evaluate differences in survival curves. Univariate and multivariate prognostic analyses were performed using COX proportional regression models. P < 0.05 was considered statistically significant.

## Results

### PTK6 protein is highly expressed in the LNM + group

**Fig. 1:**
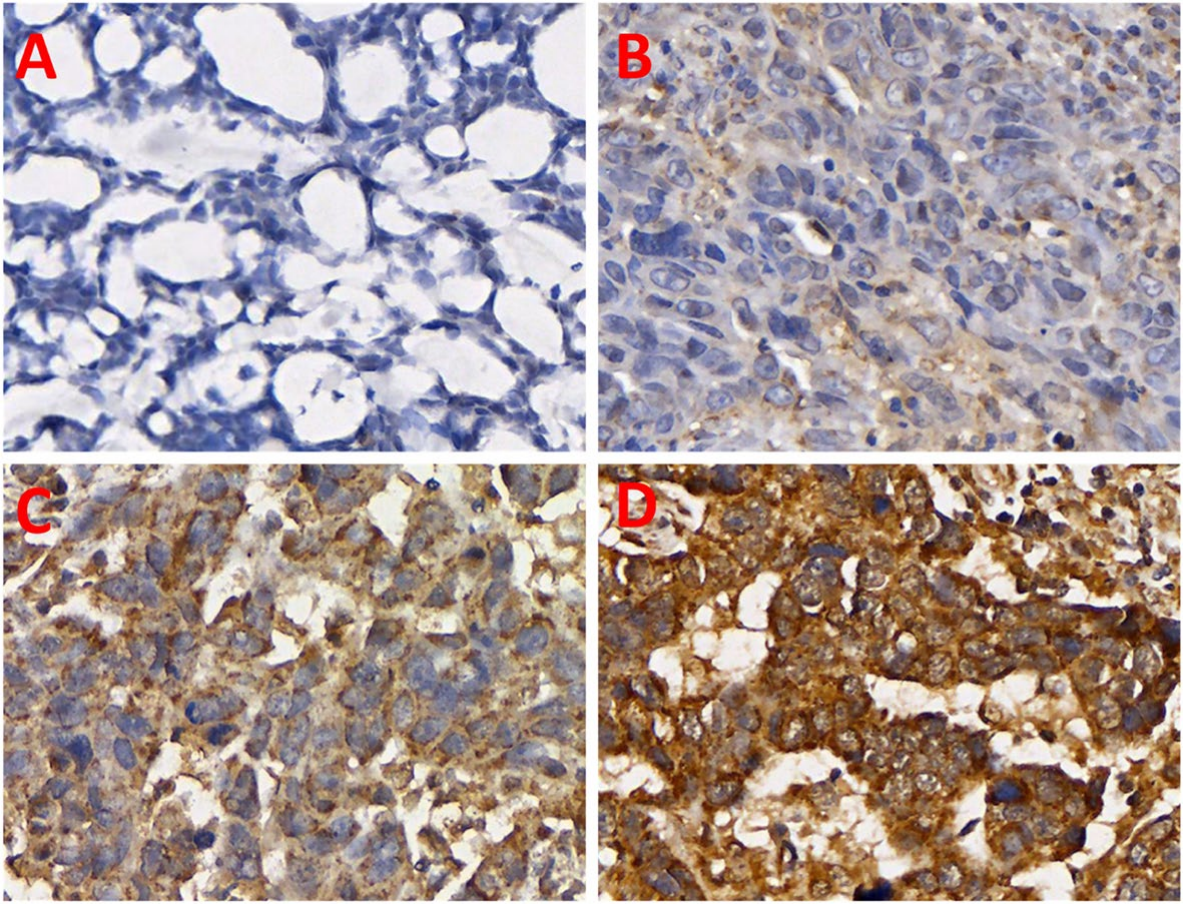
PTK6 staining strength (400X) **A** 0(no staining); **B** 1(weak staining, yellow–brown); **C** 2(medium staining, yellow–brown); **D** 3 (Strong stain, brown)

**Fig. 2:**
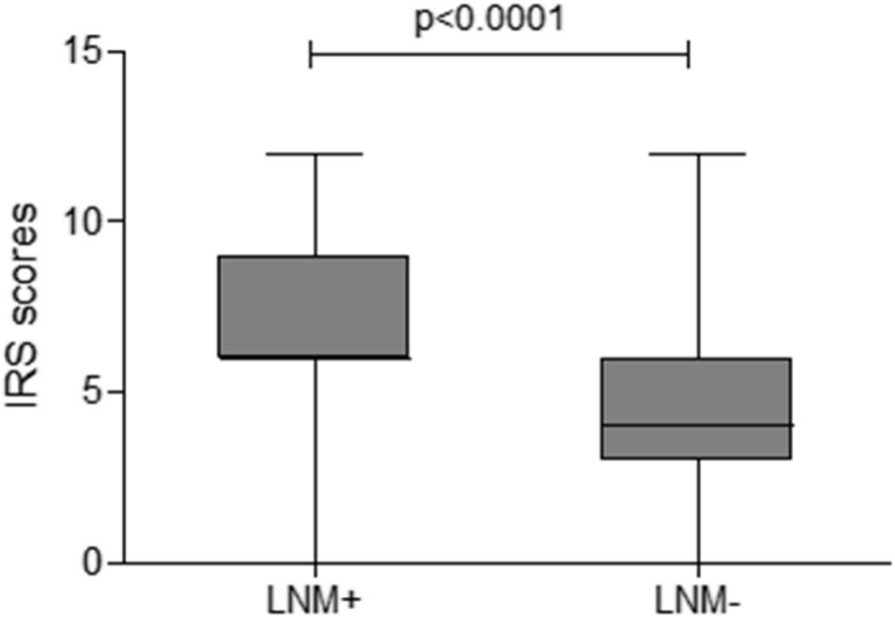
IRS scroes of different groups LNM + represented lymph node metastasis group; LNM- represented no lymph node metastasis group

**Fig. 3:**
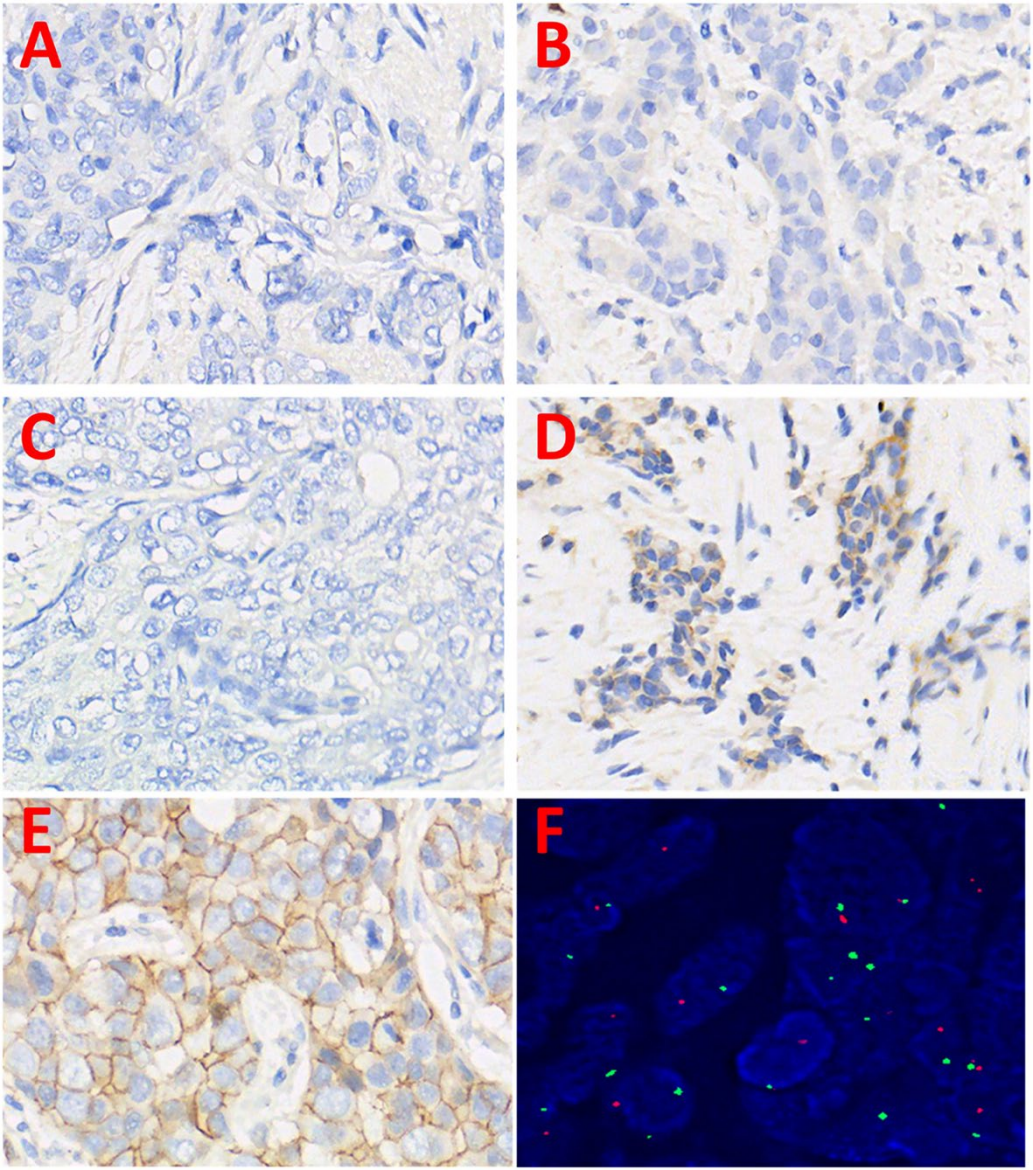
Immunohistochemical Analysis of ER,PR,HER2(400X). **A** ER negative; **B** PR negative; **C** HER2Immunohistochemical score 0: No staining on tumor cellmembrane; **D** HER2 Immunohistochemical score 1 + : Faintly perceptible staining on > 10% tumor cell membrane **E** HER2 Immunohistochemical score 2 + : Moderate staining on > 10%tumor cell membrane; **F** HER2 FISH,Low-level amplification of HER-2

The cancer tissues of all TNBC patients were examined for PTK6 expression using IHC technique. The results of this analysis are shown in Table 1, and an example of the staining is shown in Fig. 1. The incidence of PTK6 expression in the LNM + group was 78.4% and in the LNM-group it was 28.0% (*P* < 0.05), suggesting that PTK6 protein expression may be related to lymph node metastasis. The IRS score for PTK6 in the LNM + group was also higher than in the LNM- group (*P* < 0.0001), as shown in Fig. 2. This result suggests that PTK6 expression may be correlated with tumor cell metastasis. Since many previous studies have reported that PTK6 promotes tumor cell migration [15], PTK6 is more likely to promote triple-negative breast cancer metastasis. The results of triple negative IHC staining of breast cancer are shown in Fig. 3.

**Table 1:** PTK6 protein expression in different groups

PTK6	LNM +	LNM-	*P* value
**Negative(IRS: 0–4)**	22	77	< 0.05
**Positive(IRS: 5–12)**	80	30	
**Total**	102	107	

LNM + represented lymph node metastasis group; LNM- represented no lymph node metastasis group

### PTK6 protein expression and clinicopathologic features of TNBC

Table 2 shows the correlation analysis between PTK6 protein expression and various clinicopathological features of TNBC patients, including histological type, age, site, histological grade, T stage, N stage, distant metastasis or recurrence, and overall survival.

**Table 2:** PTK6 protein expression and clinicopathologic features

**Clinicopathologic features**	**LNM + **		***P***	**LNM-**		***P***
	**-**	** + **		**-**	** + **	
**Histological type**			0.451^b^			0.498^a^
IDC	20	65		54	19	
Other types	2	15		23	11	
**Age(y)**			0.368^a^			0.155^a^
≥ 50	10	45		40	11	
< 50	12	35		37	19	
**Site**			0.656^a^			0.427^a^
Left breast	13	43		50	17	
Right breast	9	37		27	13	
**Histological grading**			0.308^c^			0.312^c^
I	1	0		0	0	
II	6	25		30	20	
III	15	55		40	17	
**T stage**			0.416^b^			0.181^b^
T1-T2	21	69		74	26	
T3-T4	1	11		3	4	
**N stage**			0.322^b^			N
N1	16	49		N	N	
N2	4	12		N	N	
N3	2	19		N	N	
**HER2**			0.917^a^			0.711^a^
N	11	39		38	16	
L	11	41		39	14	
**D or R**			0.078^b^			1^b^
Yes	3	29		8	3	
No	19	51		69	27	
**Survival time(y)**			0.018^c^			N^c^
< 5	0	15		0	0	
≥ 5	22	55		79	30	

Represented PTK6 negative(IRS: 0–4); + represented, *PTK6* Positive(IRS: 5–12), *IDC* Represented invasive ductal Carcinoma; Other types represented specific types of invasive ductal carcinoma; HER N represented HER2 negative; HER L represented HER2 low expression of HER2; D or R distant represented metastasis or recurrence. N represented not detected. P represented *P* value

^a^Pearson Chi-Square

^b^Continuity Correction

^c^Fisher's Exact Test

PTK6 protein expression in the LNM + group correlated with 5 years overall survival, and that patients with high PTK6 expression had worse prognosis.

However, there were no statistically significant associations between PTK6 protein expression and any of the clinical parameters in the LNM- group.

### No correlation between HER2- low expression and PTK6 protein in the TNBC groups

HER2-low breast cancer refers to tumors with low levels of HER2 protein expression and no detectable gene amplification (IHC1 + or IHC2 + and FISH negative). Studies on the prognosis of HER2-low breast cancer have yet to reach consensus [24–26]. These account for 45–55% of all breast cancer patients, and 54% in Chinese breast cancer patients [24, 27].

The relationship between HER2- low expression and PTK6 expression is still unclear. In the present study, patients with HER2- low expression accounted for 51% of the LNM + group, and 51% of the LNM-group also. PTK6 expression did not correlate with HER2- low expression in any of the groups(shown in Table 3), suggesting the synergistic expression of PTK6 protein and HER2 may not be applicable to patients with HER2-low expression.

**Table 3:** Correlation between HER2- low and PTK6 protein in different groups

PTK6	LNM +		*P*	LNM-		*P*
	HER2-L	HER2-N	0.9	HER2-L	HER2-N	0.7
**-**	11	11		39	38	
** + **	41	39		14	16	

represented PTK6 negative(IRS: 0–4); + represented PTK6 positive(IRS: 5–12);HER2-L represents HER2- low, HER2-N means HER2-negative;LNM + represented lymph node metastasis group; LNM- represented no lymph node metastasis group.P represented *P* value

### High PTK6 protein expression is associated with poor prognosis of TNBC patients in the LNM + group

**Fig. 4:**
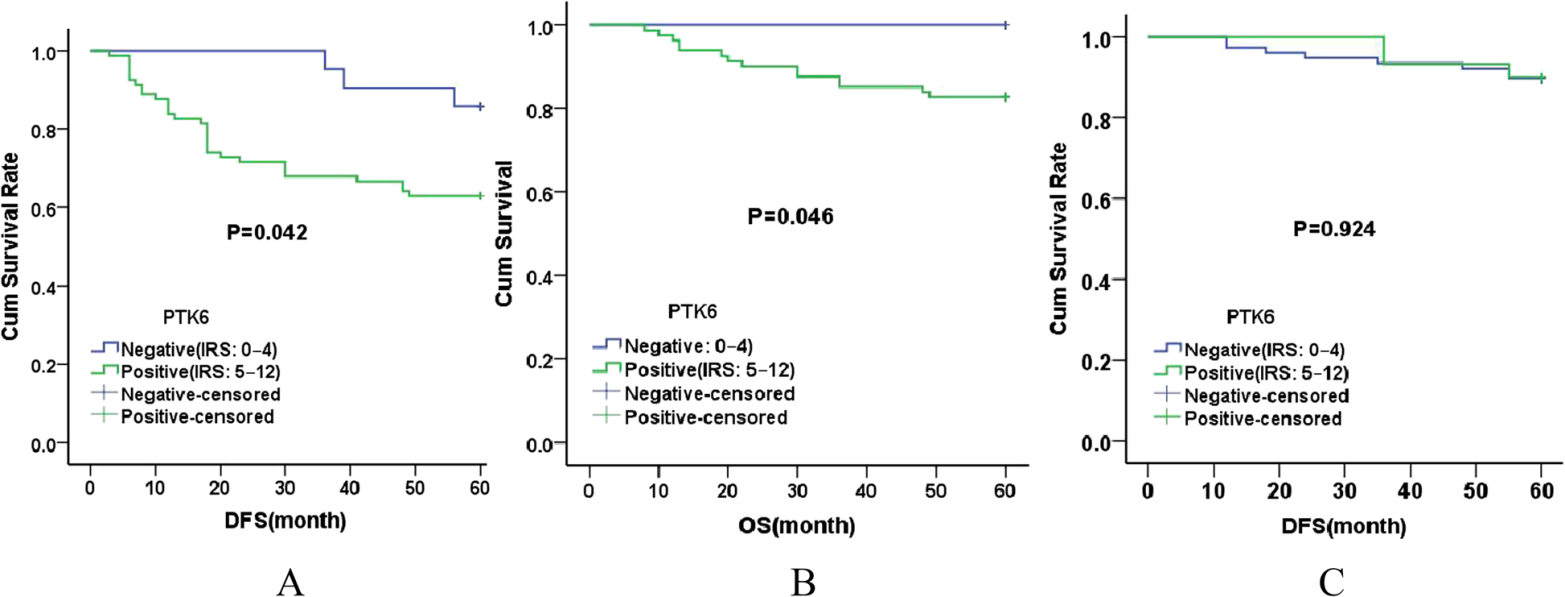
Kaplan–Meier analysis of DFS and OS in different groups **A**. DFS of lymph node metastasis group(LNM +), **B**. OS of lymph node metastasis group(LNM +), **C**. DFS of no lymph node metastasis group(LNM-)

In the current study, Kaplan–Meier survival analysis revealed that high PTK6 protein expression was associated with worse OS(*P* = 0.046)and DFS(*P* = 0.042)in the LNM + group (Fig. 4). These results suggest that inhibition of PTK6 may be a potential target for TNBC therapy. Wang et al. also found that PTK6 was associated with poor prognosis of breast cancer [28]. PTK6 expression was not associated with DFS in the LNM- group.

Subsequently, we conducted COX survival analysis, as shown in Table 4. In the LNM + group, COX univariate and multivariate analysis showed that PTK6 expression(*P* = 0.039,HR = 4.507) and N stage(*P* = 0.011,HR = 2.432) were independent risk factors for DFS. However, no risk factors affecting overall survival were found. In the LNM- group, low HER2 expression(*P* = 0.049,HR = 0.215) was a risk factor for DFS, but not an independent risk factor.

**Table 4:** COX survival analysis in different groups

parameter	LNM +				LNM-	
	OS	*P*	DFS	*P*	DFS	*P*
	HR(95%CI)		HR(95%CI)		HR(95%CI)	
**COX univariate survival analysis**						
PTK6	28.957 (0. 115–7280.0)	0.233	4.665(1.116–19.507)	0.035	0.937(0.249–3.534)	0.924
Age(y)	3.454 (0.963–12.385)	0.057	1.951(0.946–4.026)	0.070	0.925(0.282–3.032)	0.898
Histological type	0.881(0.197–3.937)	0.868	0.922(0.356–2.389)	0.868	1.188(0.348–4.059)	0.783
HER2	0.719(0.249–2.072)	0.541	0.887(0.448–1.755)	0.730	0.215(0.046–0.994)	0.049
T stage	3.132(0.982–9.989)	0.054	1.949(0.804–4.722)	0.139	0.045(0.000–3138.2)	0.587
N stage	4.853(1.521–15.482)	0.008	2.498(1.257–4.967)	0.009	None	None
breast	1.896(0.653–5.658)	0.252	1.314(0.662–2.607)	0.435	0.955(0.279–3.262)	0.941
Histological grading	1.374(0.383–4.927)	0.625	1.479(0.642–3.407)	0.358	0.727(0.222–2.381)	0.598
**COX multifactor survival analysis**						
PTK6			4.507(1.077–18.856)	0.039		
N stage			2.432(1.223–4.839)	0.011		

HER2 represents HER2- low or not; breast represents Left breast or Right breast; LNM + represented lymph node metastasis group; LNM- represented no lymph node metastasis group.P represented P value;OS represented Overall survival, DFS represented Disease-free survival

## Discussion

TNBC is a highly aggressive form of breast cancer, accounting for about 10–20% of all cases and being more common in young women.Systemic cytotoxic chemotherapy regimens, including taxanes, are currently used to treat TNBC.However, chemotherapy resistance often develops and hence there is an urgent need to identify novel molecular targets for other drugs. In the early years, Some scholars crossed Ptk6-/- mice with the mouse mammary tumor virus-ERBB2 transgenic mouse line expressing activated ERBB2 and characterized by tumor development and progression, the role of PTK6 in ERBB2-induced breast tumor initiation and metastasis was explored [20]. PTK6 has also been shown to promote cell proliferation and migration through phosphorylation of Eps8, regulate the expression of E-cadherin through SNAIL protein degradation, promote epithelial mesenchymal transformation and regulate the metastasis of triple-negative breast cancer cells [14, 15]. PTK6 expression has been shown to contribute to breast cancer cell migration, invasion and metastasis in many other studies [14, 29–32].

Although PTK6 plays an important role in tumor development, it can act as a promoter or inhibitor depending on the tumor type [33, 34]. Hsieh et al. reported that up-regulation of PTK6 through epigenetic modification promoted the proliferation, migration, and invasion of oral squamous cell carcinoma cells [35]. These authors also found that PTK6 promoted the development and metastasis of cancer by increasing STAT3 phosphorylation and ZEB1 expression. In a study of prostate cancer, activation of PTK6 in the plasma membrane was found to increase phosphorylation and to activate AKT,p130Cas and FAK, thereby promoting cancer cell proliferation, survival, migration, and epithelial-mesenchymal cell transformation [11].

PTK6 has also been found to induce tumor cell migration, invasion and metastasis in breast cancer, ovarian cancer, nasopharyngeal cancer, small cell lung cancer and prostate cancer [36–39]. However, PTK6 appears to promote apoptosis in colorectal cancer, human osteosarcoma U2OS cells and astrocytes, thereby inhibiting cell division and proliferation [40–42]. However, the mechanism of this difference remains unclear and may be due to differences in tumor type, sample size, and statistical methods.PTK6 regulates RhoA and Ras via phosphorylation of p190 to promote the growth, migration and invasion of breast cancer [43]. It thus has important prognostic value in this cancer type.Results obtained recentlyusingmouse models have shown that PTK6 promotes cell survival, delays degeneration, and favors tumor formation by inducing a P38-driven pro-survival signaling pathway. These studies strongly suggest a role for PTK6 in the promotion of cell proliferation and migration [8]. Hypoxia inducible factor (HIF) 1α/2α, glucocorticoid receptor (GR), and the phosphorylated S134-GR/PELP1/HIF signaling complex have been shown to induce the expression of PTK6, thus promoting the survival and metastasis of tumor cells [29, 44, 45]. Moreover, PTK6 binds to EGFR to enhance pro-mitosis signaling by promoting the recruitment of phosphatidylinositol 3-kinase (PI3K) and activating Akt. This stimulates cell migration by activating signaling molecules such as mitogen activated protein kinase (MAPK) and parsiline [46]. In the present study, the incidence of PTK6 positive expression in the LNM + subgroup (78.4%) of TNBC patients was significantly higher than in the LNM- subgroup (28%). This result suggests that PTK6 may be related to tumor metastasis, but the relationship between these two need to be revealed by more experiments in the future. However, combined with the above studies, PTK6 is more likely to promote the metastasis of triple-negative breast cancer.

Breast tumor kinase (Brk/PTK6) was overexpressed in over 80% of breast cancers and is associated with poor patient outcomes.PTK6 protein expression is associated with different prognosis indifferent tumor types. Xu et al. found that PTK6 overexpression correlated with poor prognosis in patients with bladder cancer [47]. However, high PTK6 expression was associated with good OS and DFS in laryngeal squamous cell carcinoma patients [33]. The mechanisms responsible for these differences between tumor types is presently unclear. In the current study, Kaplan–Meier survival analysis showed that the OS and DFS of PTK6-positive patients were lower than those PTK6-negative patients in the LNM + group, which also confirmed that PTK6 was associated with poor prognosis of breast cancer. However, PTK6 protein expression did not show statistical significance for DFS in the LNM- group. Subsequently, COX survival analysis was performed, and the results of COX univariate and multivariate analysis showed that PTK6 protein expression(*P* = 0.039,HR = 4.507) and N stage(*P* = 0.011,HR = 2.432) were independent risk factors for DFS, and PTK6-positive patients were more likely to develop tumor progression, recurrence or metastasis than that PTK6-negative patients in the LNM + group, representing a poor prognosis. Patients with higher N stages are also more likely to have tumor progression, recurrence, or metastasis,also. However, no risk factors affecting OS were found in the LNM + group, which may be due to the fact that 5 years as the cut-off point for observing survival influenced the experimental results. However, in the LNM- group, only HER2-low expression(*P* = 0.049,HR = 0.215) was observed as a risk factor for DFS, but not an independent risk factor. The inconsistent survival analysis results of the LNM + group and LNM- group may be due to the different biological functions and prognostic value of PTK6 in TNBC patients with different lymph node metastasis status, or perhaps PTK6 is only negatively associated with the prognosis of patients with metastatic triple-negative breast cancer, or it may also be related to the low positive rate of PTK6 in the LNM- group, and the limited ability to promote tumor metastasis and recurrence. More experiments may be needed in the future to uncover this phenomenon.PTK6 expression has been studied in different breast cancer subtypes. Up-regulation of PTK6 expression can promote the growth of ER + breast cancer cells, whereas down-regulation can induce cell apoptosis. These results suggest that PTK6 may be involved in regulating the growth and survival of drug-resistant ER + breast cancer cells and with drug resistance to endocrine therapy, thereby supporting combined ER/PTK6 targeting of ER + breast cancer [48, 49]. Several studies have reported that PTK6 and c-erbB-2 protein expression in breast cancer is positively correlated, suggesting they play a synergistic role in the occurrence and development of breast cancer, and therefore it has been suggested that inhibition of both these signaling proteins may be more effective in the control of breast cancer [20, 50–53]. Therapy for breast cancer patients with low HER2 expression has become a hot topic in recent years. Novel HER2 antibody–drug conjugates(ADCs) may benefit such patients.In the present study, however, PTK6 protein expression did not correlate with the expression of HER2. This may be related to the expression status of HER2, since most previous studies were performed on patients with HER2 overexpression, whereas most of the patients in the present study had HER2-low or HER2- negative expression. Hence, combined PTK6/HER2 inhibition may not be suitable for patients with HER2-low or HER2- negative expression.

A role for PTK6 in the occurrence and development of tumors appears to have been confirmed. Consequently, many studies have focused on inhibiting PTK6 expression in order to slow tumor progression.The novel compounds(E)-5-(benzylideneamino)-1 h-Benzo [d]imidazol-2(3H), semi-synthetically optimized sipholenol A ester, and 4-aniline-substituted α-carboline have all been confirmed to prevent tumor development by inhibiting the expression of PTK6 [17–19]. Other studies have also shown that Hsp90 plays an important role in regulating the stability of PTK6, raising the possibility that Hsp90 inhibitors may be used as therapeutic drugs for PTK6-positive cancers,including breast cancer [54]. While it is clear that PTK6 can promote the proliferation and migration of tumor cells, the specific mechanism and signaling pathway is still unknown. Although many inhibitors have been designed for BRK, none have reached the clinical stage. Understanding the successes and challenges involved in the development of these inhibitors is critical for producing clinically relevant drugs in the future [55]. Roja et al. recently described the therapeutic significance of Brk and mTOR and their associated signals in the development of breast cancer, thus providing a possible new strategy for gene therapy in this cancer type [2].

This study also has some limitations, because the prognosis of breast cancer is generally good, so the 5-year survival time as the follow-up cut-off point may be a little short, setting the 10-year survival time for the study may have greater differences. The sample size will also affect the research results, and a large enough sample size can draw more accurate conclusions. In addition, the results of immunohistochemistry test and FISH test vary according to the laboratory environment, lymph node tests may show false negative results, thus affecting the grouping of experiments. All of these factors could have influenced the results of the experiment.

## Conclusion

In summary, current study showed that the positive rate of PTK6 in the LNM + group was higher than that in the LNM-group, and the former had worse OS and DFS. COX survival analysis also showed that the PTK6 expression and N stage were independent risk factors for DFS in the LNM + group. These results suggest that PTK6 positive is associated with poor prognosis and plays an important role in promoting the recurrence and metastasis of TNBC, but these results are based on the presence of lymph node metastasis in patients. Perhaps the status of lymph node metastasis in patients with TNBC can affect the effect of PTK6 on the prognosis, or perhaps PTK6 is only negatively correlated with the prognosis of patients with metastatic TNBC. In view of the poor prognosis of PTK6 for TNBC, the future research direction may be to achieve the treatment of TNBC by inhibiting PTK6, especially for patients with metastatic TNBC, and provide more drug options for patients with TNBC.

## Data Availability

All data are included in the manuscript.

## Abbreviations

PTK6: Protein tyrosine kinase 6
BRK: Breast tumor kinase
ER: Estrogen Receptor
PR: Progesterone receptor
HER2: Human epidermal growth factor receptor-2
IHC: Immunohistochemical
FISH: Fluorescence in situ hybridization
TNBC: Triple negative breast cancer
OS: Overall survival
DFS: Disease-free survival
